# Associations between adipokines gene polymorphisms and knee osteoarthritis: a meta-analysis

**DOI:** 10.1186/s12891-022-05111-4

**Published:** 2022-02-22

**Authors:** Yuqing Wang, Fanqiang Meng, Jing Wu, Huizhong Long, Jiatian Li, Ziying Wu, Hongyi He, Haochen Wang, Ning Wang, Dongxing Xie

**Affiliations:** 1 Department of Orthopaedics, Xiangya Hospital, Central South University, Changsha, Hunan China; 2 Hunan Key Laboratory of Joint Degeneration and Injury, Xiangya Hospital, Central South University, Changsha, China; 3 Hunan Engineering Research Center for Osteoarthritis, Changsha, China; 4 National Clinical Research Center for Geriatric Disorders, Xiangya Hospital, Central South University, Changsha, China

**Keywords:** Meta-analysis, Adipokines, Knee osteoarthritis, Polymorphisms

## Abstract

**Background:**

Adipokines gene polymorphisms are speculated to be associated with the risk of knee osteoarthritis (OA), but evidence remains conflicting. This study therefore aimed to examine whether associations exist between adipokines gene polymorphisms and knee OA by considering the evidence collected from eligible studies through a meta-analysis.

**Methods:**

A systematic search was performed on PubMed, Embase, Web of Science, China National Knowledge Infrastructure (CNKI), and Wanfang up to March 31, 2020. Meta-analysis was carried out by focusing on the associations between adipokines gene polymorphisms and knee OA with the allele model, dominant model, and recessive model.

**Results:**

The present meta-analysis included 5 eligible studies for ADIPOQ rs1501299 with 1,021 cases and 1,097 controls, 3 eligible studies for ADIPOQ rs2241766 with 549 cases and 544 controls, 3 eligible studies for LEPR rs1137101 with 808 cases and 856 controls, 2 eligible studies for VISFATIN rs4730153 with 339 cases and 680 controls and 2 eligible studies for VISFATIN rs16872158 with 339 cases and 680 controls. Significant association was observed between LEPR rs1137101 and knee OA in the overall population (recessive: OR = 0.40, 95% CI 0.21–0.79). Limited data revealed that associations may exist between ADIPOQ rs2241766 and knee OA in Asians (dominant: OR = 1.35, 95% CI 1.03–1.78), between VISFATIN rs4730153 and knee OA in Asians (allele: OR = 0.58, 95% CI 0.41–0.83; dominant: OR = 0.57, 95% CI 0.39–0.83), and between VISFATIN rs16872158 and knee OA in Asians (allele: OR = 1.84, 95% CI 1.26–2.68; dominant: OR = 1.94, 95% CI 1.31–2.89).

**Conclusions:**

Adipokines gene polymorphisms may be associated with knee OA. The association was observed in LEPR rs1137101 in the present study. In addition, limited data revealed that associations may also exist in ADIPOQ rs2241766, VISFATIN rs4730153 and VISFATIN rs16872158.

**Prospero registration:**

CRD42020187664.

**Supplementary Information:**

The online version contains supplementary material available at 10.1186/s12891-022-05111-4.

## Background

Osteoarthritis (OA), a highly prevalent disease, was estimated to affect 250 million people worldwide at present [1], and has therefore become a major contributor to global disability [2]. OA is characterized by degeneration of articular cartilage, synovial inflammation and bone remodeling, which can consequently lead to pain, physical activity limitations and markedly reduced quality of life [3]. At present, pharmacological treatment options for OA lack approved disease-modifying therapies and are largely limited to the relief of symptoms [4], for end-stage OA patients, joint replacement is demanded [5]. Elucidation of the underlying etiology of OA would be helpful for confirming diagnosis in early stages, therefore facilitating timely and effective clinical decision making. OA has a multifactorial pathophysiology, which may involve mechanical, metabolic and inflammatory contributors [6]. In addition, genetic factors may explain a large part of the susceptibility to OA [7]. In the past few years, several studies reported OA risk loci have been published [8–12], including genome-wide association studies which discovered DNA variants, primarily the single nucleotide polymorphisms (SNPs) in large cohorts [12]. Insights from these relevant studies have firmly placed OA into the polygenic category of common diseases [13–16].

The recognized prominent risk factors of OA include increasing age, female sex and obesity [4]. In particular, obesity is a well-established risk factor [4] due to its potential contribution in the mechanical aspect by increasing the joint load [17], as well as in the metabolic aspect by playing the role of adipose tissue as an endocrine organ secreting a variety of metabolically-active mediators. Among these secreted mediators, adipokines are a main type [17]. Indeed, adipose tissue has been confirmed to release an array of adipokines including adiponectin, leptin, resistin, and visfatin [17, 18], among which leptin was first discovered by Friedman et al. in 1994 [19]. Then, in 2003, Dumond et al. [20] derived the earliest evidence supporting a pivotal role of leptin in OA. This milestone study initiated the journey to examine adipokines as a possible metabolic link between obesity and OA.

Knee is the most common site in OA [1], Several SNPs of the adipokines genes have been associated with knee OA [21–32], but the results are inconsistent. For instance, a study from Thailand rejected any significant association between the ADIPOQ gene rs1501299 polymorphism and knee OA [24], while Jin et al. reported that rs1501299 polymorphism intensified the risk of knee OA in Chinese subjects.

[21]. In view of the limitations in deriving comprehensive conclusions from individual studies and the inconsistency among different studies, we intended to conduct a meta-analysis to clarify whether the main types of adipokines gene SNPs could be associated with the susceptibility to knee OA.

## Methods

### Search methods

The PubMed, Embase, Web of Science, China National Knowledge Infrastructure (CNKI), and Wanfang databases were searched through to retrieve observational studies that focused on the associations between adipokines gene polymorphisms and OA up to March 31, 2020 (Appendix). The present meta-analysis has been conducted according to the Preferred Reporting Items for Systematic Reviews and Meta-analyses (PRISMA) reporting guideline (Additional file 1).

### Inclusion and exclusion criteria

Two investigators assessed the retrieved studies independently according to the pre-specified inclusion criteria as follows: (1) knee OA was diagnosed based on the American College of Rheumatology criteria or radiographic findings, or the patient received total joint replacement because of primary knee OA; (2) observational studies that investigated the associations between adipokines gene polymorphisms and knee OA; (3) observational studies that compared knee OA patients with healthy controls; (4) the allele and genotype distributions of healthy controls were compliant with the Hardy–Weinberg equilibrium (HWE) model; (5) the frequency distributions of alleles and genotype were available. The exclusion rules were: (1) duplicated publications; (2) conference abstracts or commentaries; (3) animal or in vitro studies; (4) review articles.

### Data extraction and evaluation of study quality

Two investigators extracted the desired data (i.e., authors, publication year, study design, country, OA site, genotype method, sample size of the case group and control group, and the allele and genotype frequency of adipokines SNPs) from eligible studies independently.

Another two investigators analyzed the methodological quality of each study independently by applying the Newcastle–Ottawa Scale (NOS) [33], in terms of the selection of study participants, comparability of outcome groups and outcome measures. A NOS score > 6 indicated a high-quality study [34, 35].

### Statistical analysis

The conformity of the distributions of observed allele or genotype frequencies to HWE in the control group was verified by chi-square test. The statistical heterogeneity was tested by *I*^2^ statistics. The odd ratios (ORs) and 95% confidence intervals (CIs) were estimated by the random effects model in case of high heterogeneity (*I*^2^ > 50%), and by the fixed effects model in case of low heterogeneity [36]. Publication bias was examined by the Begg’s test [37] and the Egger’s test [38], where *P* < 0.05 implied statistical significance. All data analyses were performed in Stata 15.0 (Stata Corp, College Station, TX, USA).

The meta-analysis was carried out on (1) the allele model, (2) the dominant model, and (3) the recessive model. In order to examine the effects on specific demographic regions, subgroup analyses were performed on each of the different populations. Then, sensitivity analyses were performed to evaluate the impact of any single study on the overall effects by examining the ORs alongside their matching 95% CIs before and after eliminating each study from the meta-analysis.

## Results

### Eligible studies

**Fig. 1:**
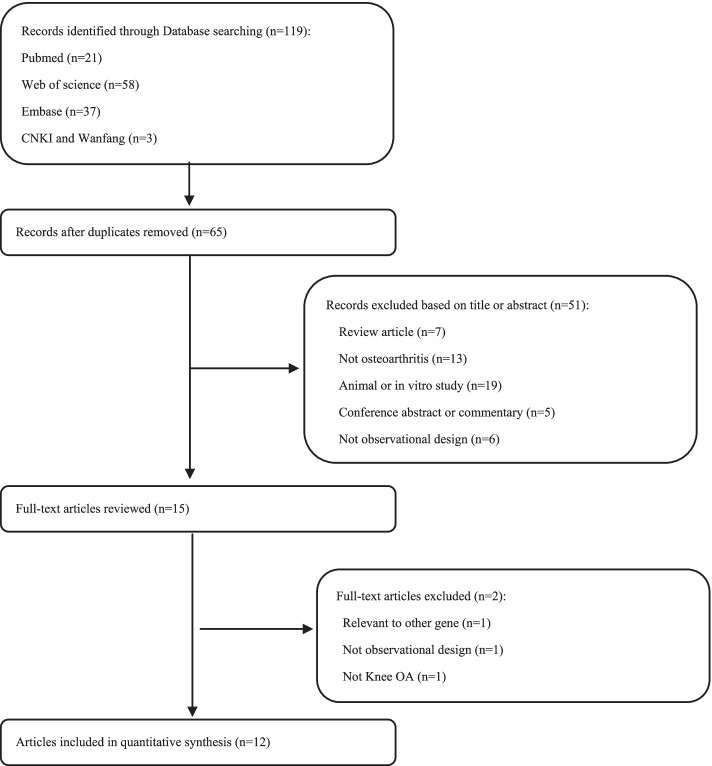
Selection process of eligible studies

Figure 1 shows the flow chart illustrating the selection process. Our meta-analysis included 5 eligible studies for ADIPOQ rs1501299 (1,021 cases and 1,097 controls), 3 eligible studies for ADIPOQ rs2241766 (549 cases and 544 controls), 3 eligible studies for LEPR rs1137101 (808 cases and 856 controls), 2 eligible studies for VISFATIN rs4730153 (339 cases and 680 controls) and 2 eligible studies for VISFATIN rs16872158 (339 cases and 680 controls) [21–32]. Tables 1 and 2 summarize the main characteristics of the studies included. The allele and genotype distributions of the control group showed consistency with the HWE. All the included studies were judged as high quality (NOS score > 6).

**Table 1:** Main Characteristics of Studies Included in This Meta-analysis

Study	Design	Country	OA site	Sample size		Mean Age (years)		Gender (M/F)		Genotyping method	Matching	NOS	Gene (SNPs)
				OA	Control	OA	Control	OA	Control				
Jin 2019	Case–control	China	Knee	372	453	50.23	51.12	158/214	199/254	PCR–RFLP	Age, sex, BMI	7	ADIPOQ (rs1501299)
Espinosa-Morales 2019	Case–control	Mexico	Knee	92	147	47.2	40.9	12/80	42/105	TaqMan	Not available	7	ADIPOQ (rs1501299, rs2241766)
Liu 2018	Case–control	China	Knee	196	442	62.19	57.17	48/148	139/303	PCR	Sex, occupation	8	ADIPOQ (rs182052, rs2082940, rs6773957)
Honsawek 2017	Case–control	Thailand	Knee	202	196	68.8	65.2	66/136	68/128	PCR–RFLP	Not available	7	ADIPOQ (rs2241766, rs1501299)
Honsawek 2014	Case–control	Thailand	Knee	100	100	68.2	67.0	25/75	20/80	PCR–RFLP	Age, sex, BMI	8	ADIPOQ (rs1501299)
Zhan 2016	Case–control	China	Knee	255	201	64.2	65.2	39/216	68/133	PCR–RFLP	Age	8	ADIPOQ (rs2241766, rs1501299)
Yang 2016	Case–control	China	Knee	587	628	61.37	60.46	134/453	143/485	PCR–RFLP	Age, sex	8	LEPR (rs1137101)
Jin 2013	Case–control	China	Knee	148	155	53.18	54.27	46/102	54/101	PCR	Age, sex	7	LEPR (rs1137101)
Doudar 2020	Case–control	Egypt	Knee	73	73	56.6	53.2	15/58	12/61	PCR–RFLP	Age, sex	7	LEPR (rs1137101)
Jiang 2010	Case–control	China	Knee	697	699	59.6	58.5	169/528	411/288	TaqMan	Age	7	LEP (rs11761556, rs12706832, rs2071045)
Murtaza 2019	Case–control	Pakistani	Knee	280	308	54.6	53.5	120/160	152/156	PCR–RFLP	Age, sex, BMI	8	RESISTIN (rs3745367, rs1862513)
Jiang 2016	Case–control	China	Knee	196	442	62.19	57.17	48/148	139/303	PCR–RFLP	Not available	8	VISFATIN (rs4730153, rs16872158, rs3801267)
Jiang 2016	Case–control	China	Knee	143	238	62.10	56.95	31/112	65/173	PCR–RFLP	Not available	8	VISFATIN (rs4730153, rs16872158)

*Abbreviations*: *OA* osteoarthritis, *NA* not available *PCR* polymerase chain reaction, *PCR–RFLP* polymerase chain reaction and restriction fragment length polymorphism, *SNP*, single nucleotide polymorphism

**Table 2:** Distribution of genotypes among cases and controls

	Study ID	Case group			Control group		
		**GG**	**GT**	**TT**	**GG**	**GT**	**TT**
**ADIPOQ** rs1501299 (G > T)	Jin 2019	174	160	37	240	182	30
	Espinosa Morales 2019	55	29	3	61	40	6
	Honsawek 2017	106	76	20	102	77	17
	Honsawek 2017	58	35	7	59	36	5
	Zhan 2016	130	101	24	103	81	17
**ADIPOQ** rs2241766 (T > G)		**TT**	**GT**	**GG**	**TT**	**GT**	**GG**
	Espinosa‑Morales 2019	56	28	3	59	42	3
	Honsawek 2017	84	93	25	96	75	25
	Zhan 2016	105	120	30	98	78	25
**LEPR** rs1137101 (G > A)		**GG**	**GA**	**AA**	**GG**	**GA**	**AA**
	Yang 2016	267	271	49	258	291	79
	Jin 2013	38	79	31	21	58	76
	Doudar 2020	36	34	3	36	28	9
**VISFATIN** rs4730153 (G > A)		**GG**	**GA**	**AA**	**GG**	**GA**	**AA**
	Jiang 2015	170	25	0	350	85	4
	Jiang 2015	124	16	1	189	43	3
**VISFATIN** rs16872158 (T > A)		**TT**	**TA**	**AA**	**TT**	**TA**	**AA**
	Jiang 2015	164	30	1	400	39	2
	Jiang 2015	119	21	1	217	18	1

### Meta-analysis results

**Fig. 2:**
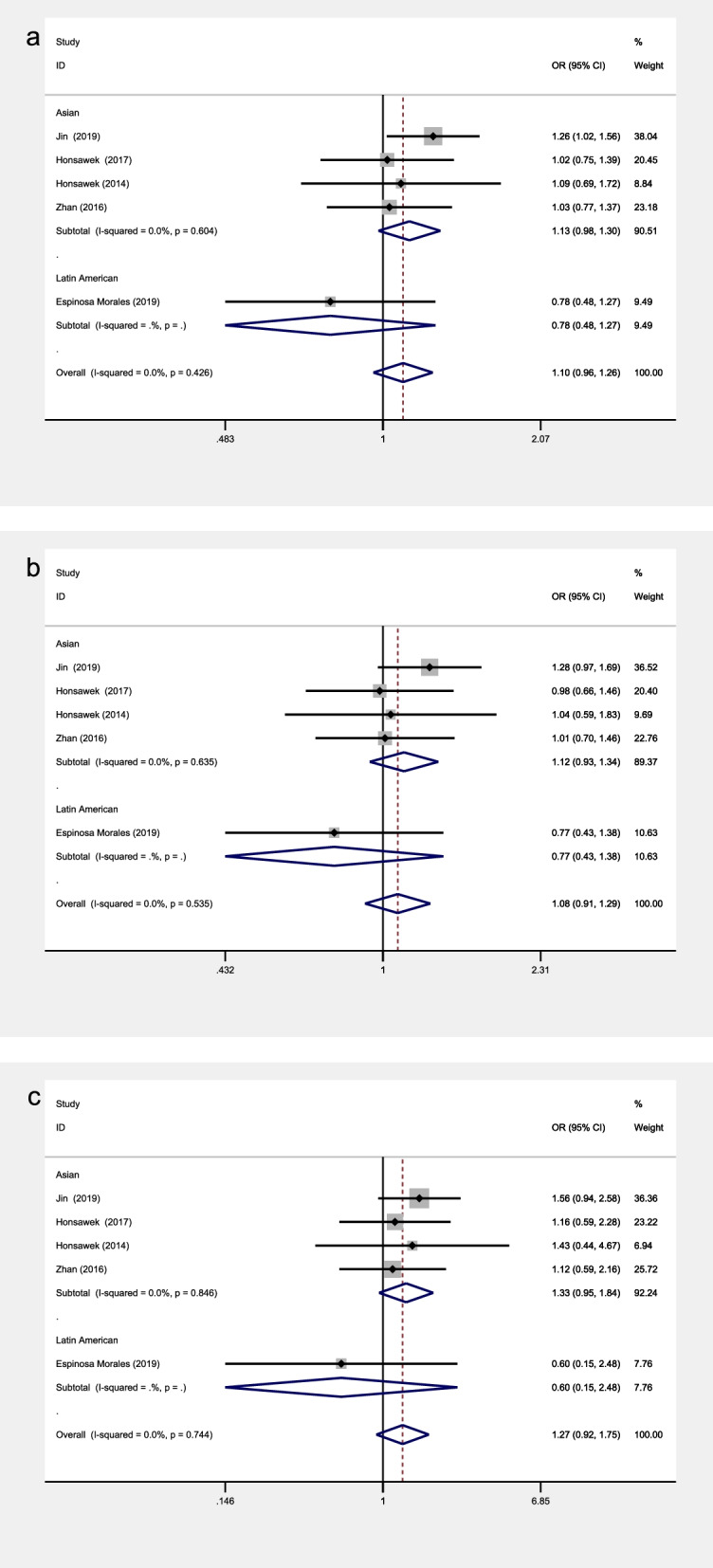
The associations of ADIPOQ rs1501299 with OA in different genetic models. **a** allele model. **b** dominant model. **c** recessive model

**Fig. 3:**
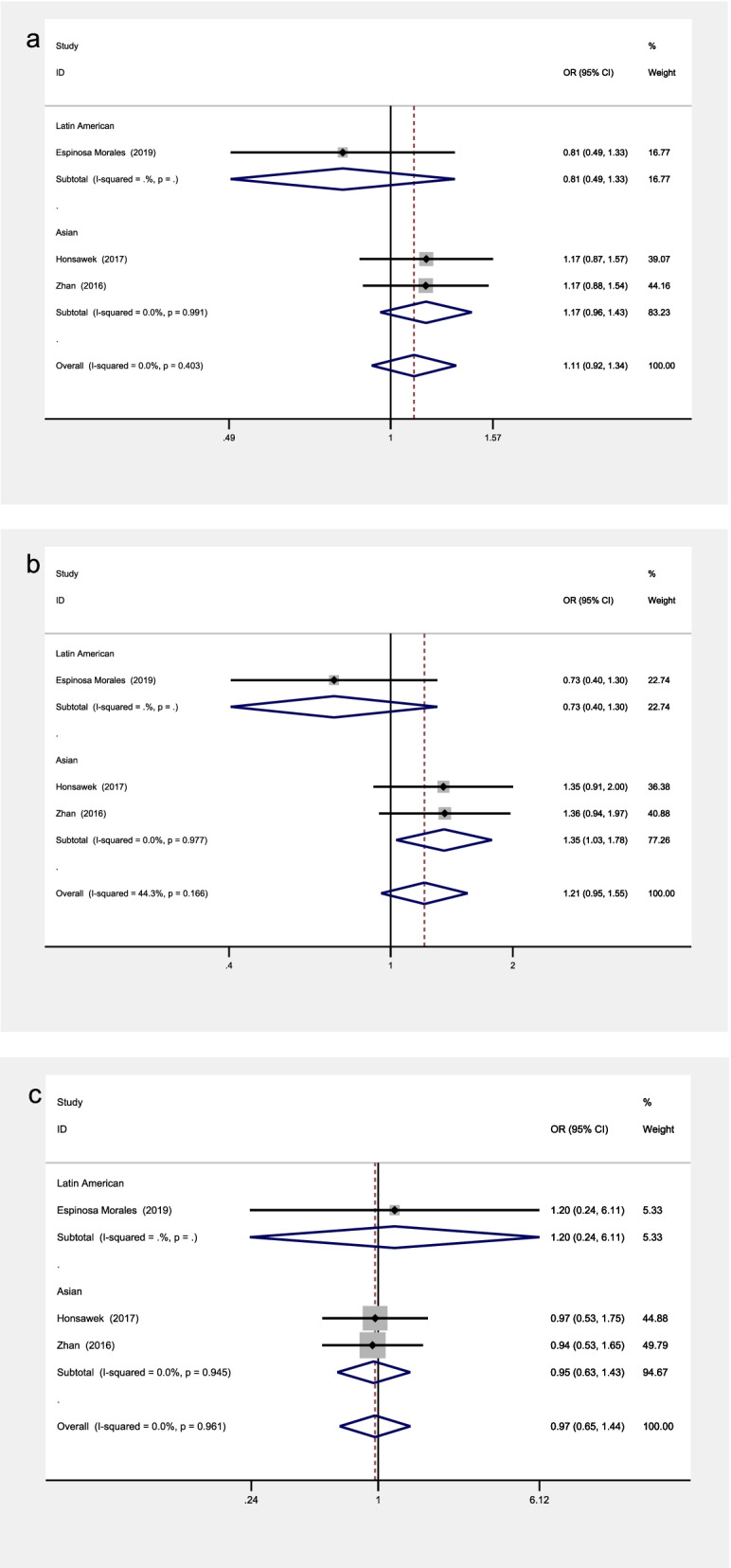
The associations of ADIPOQ rs2241766 with OA in different genetic models. **a** allele model. **b** dominant model. **c** recessive model

**Fig. 4:**
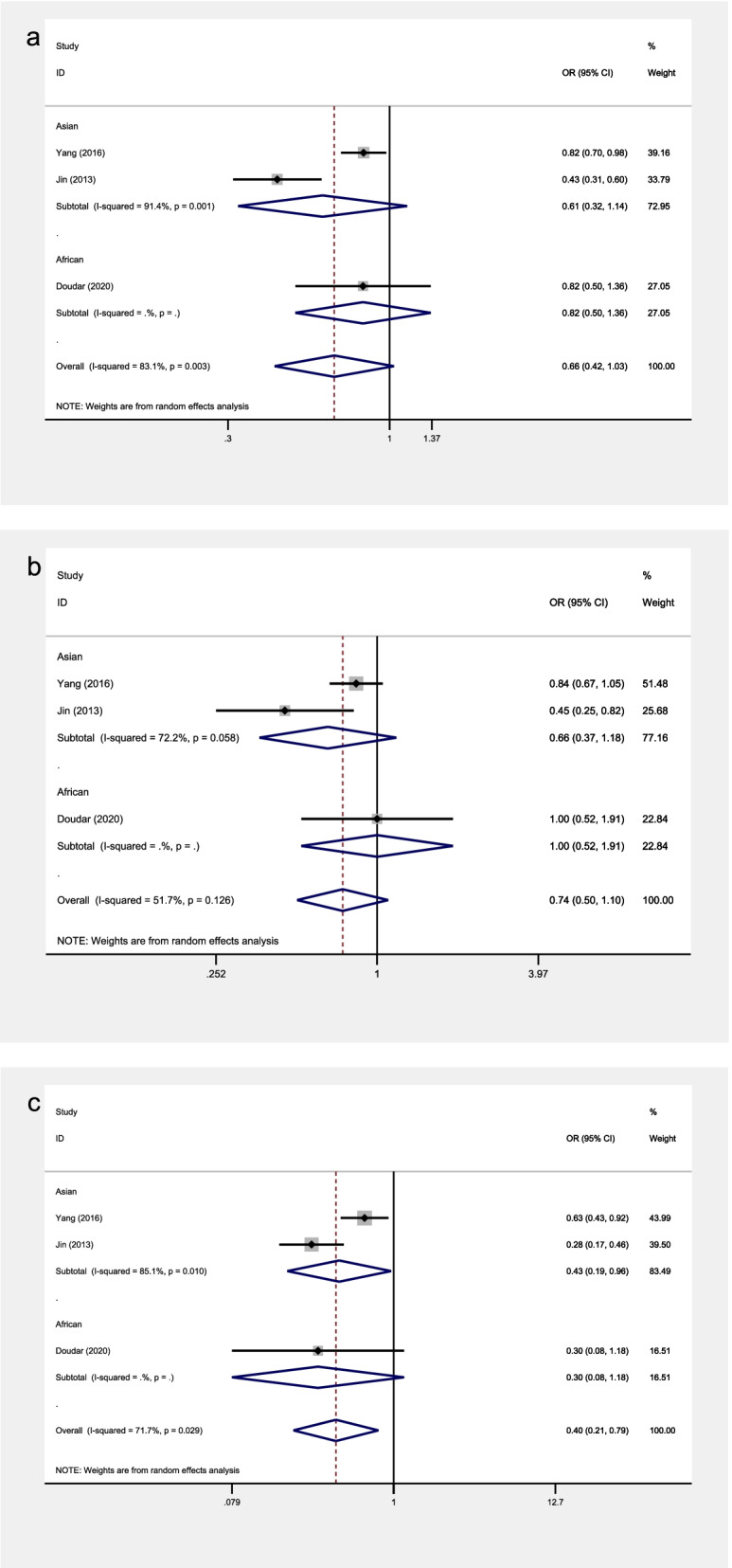
The associations of LEPR rs1137101 with OA in different genetic models. **a** allele model. **b** dominant model. **c** recessive model

**Fig. 5:**
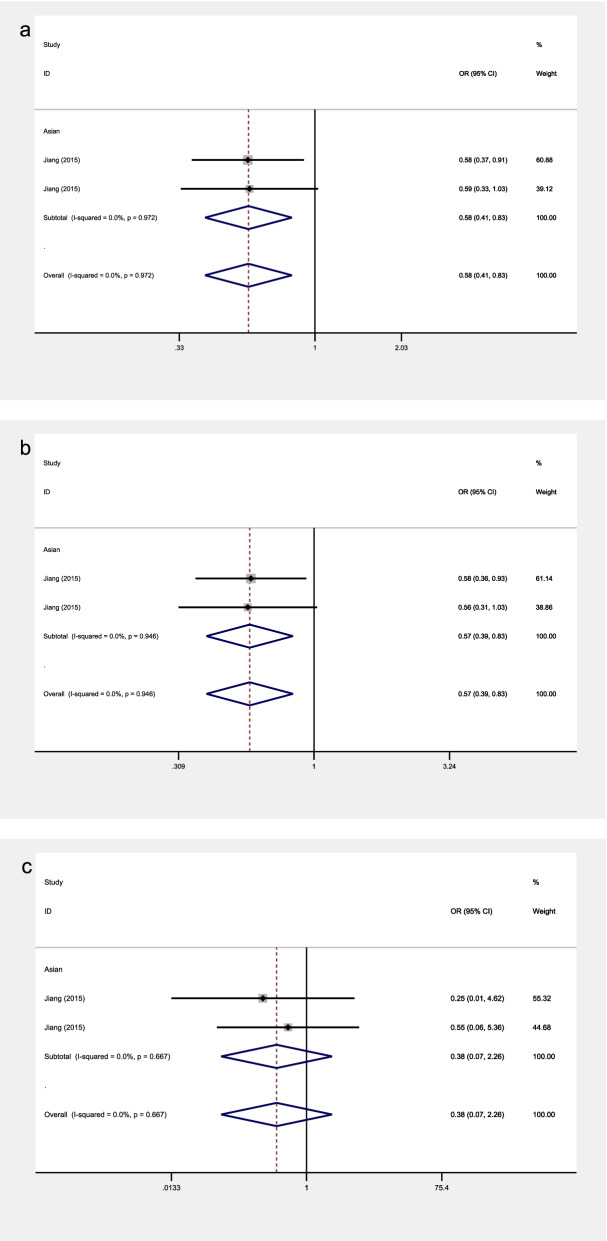
The associations of VISFATIN rs4730153 with OA in different genetic models. **a** allele model. **b** dominant model. **c** recessive model

**Fig. 6:**
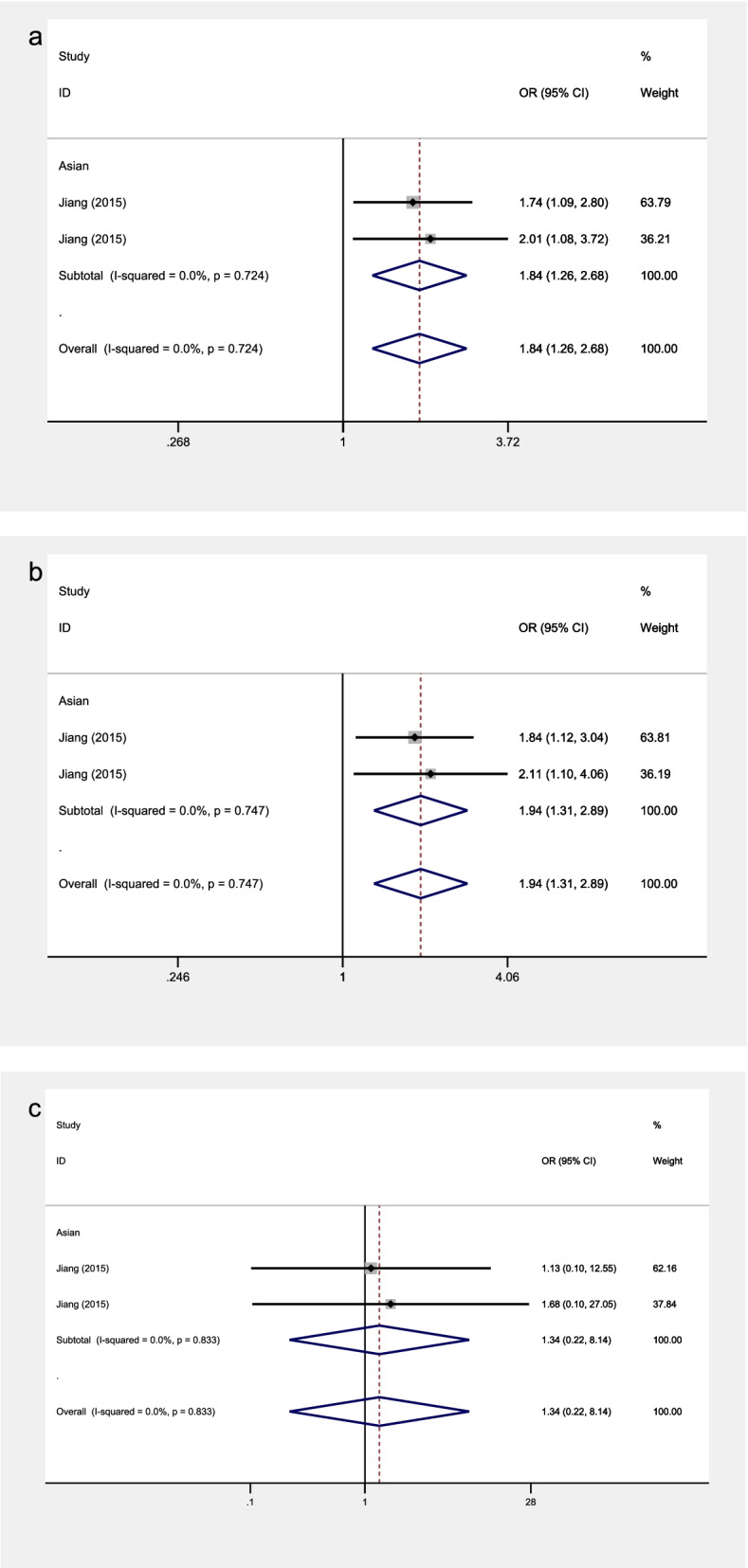
The associations of VISFATIN rs16872158 with OA in different genetic models. **a** allele model. **b** dominant model. **c** recessive model

The included studies covered a total of 14 SNPs from 5 genes, among which 5 SNPs from 3 genes were reported by at least 2 studies and were included into the meta- analysis (see Table 3 and Figs. 2, 3, 4, 5 and 6 for the main meta-analysis results). The pooled ORs and 95% CIs were calculated for the allele model, dominant model, and recessive model respectively.

**Table 3:** Meta-analysis of associations between adipokines polymorphisms and OA

Polymorphism	Population	No. of studies	Tests of association			Tests of heterogeneity			Begg’s test	Egger’s test
			OR	95% CI	*P*-value	Model	*P*-value	*I*^2^ (%)	*P*-value	*P-*value
**ADIPOQ** rs1501299 **allele**	Overall	5	1.10	0.96, 1.26	0.184	F	0.426	0	0.221	0.198
	Latin American	1	0.78	0.48, 1.27	0.326	/	/	/	/	/
	Asian	4	1.13	0.98, 1.30	0.093	F	0.604	0	/	/
**recessive**	Overall	5	1.27	0.92, 1.75	0.142	F	0.744	0	0.462	0.209
	Latin American	1	0.60	0.15, 2.48	0.481	/	/	/	/	/
	Asian	4	1.33	0.95, 1.84	0.093	F	0.846	0	/	/
**dominant**	Overall	5	1.08	0.91, 1.29	0.376	F	0.535	0	0.221	0.077
	Latin American	1	0.77	0.43, 1.38	0.381	/	/	/	/	/
	Asian	4	1.12	0.93, 1.34	0.228	F	0.635	0	/	/
**ADIPOQ** rs2241766 **allele**	Overall	3	1.11	0.92, 1.34	0.279	F	0.403	0	0.296	0.073
	Latin American	1	0.81	0.49, 1.33	0.402	/	/	/	/	/
	Asian	2	1.17	0.96, 1.43	0.130	F	0.991	0	/	/
**recessive**	Overall	3	0.97	0.65, 1.44	0.861	F	0.961	0	0.296	0.124
	Latin American	1	1.20	0.24, 6.11	0.824	/	/	/	/	/
	Asian	2	0.95	0.63, 1.43	0.813	F	0.945	0	/	/
**dominant**	Overall	3	1.21	0.95, 1.55	0.125	F	0.166	44.3	0.296	0.078
	Latin American	1	0.73	0.40, 1.30	0.283	/	/	/	/	/
	Asian	2	1.35	1.03, 1.78	0.028	F	0.977	0	/	/
**LEPR** rs1137101 **allele**	Overall	3	0.66	0.42, 1.03	0.069	R	0.003	83.1	1.000	0.677
	Asian	2	0.61	0.32, 1.14	0.120	R	0.001	91.4	/	/
	African	1	0.82	0.50, 1.36	0.441	/	/	/	/	/
**recessive**	Overall	3	0.40	0.21, 0.79	0.008	R	0.029	71.7	1.000	0.661
	Asian	2	0.43	0.19, 0.96	0.040	R	0.010	85.1	/	/
	African	1	0.30	0.08, 1.18	0.084	/	/	/	/	/
**dominant**	Overall	3	0.74	0.50, 1.10	0.137	R	0.126	51.7	1.000	0.724
	Asian	2	0.66	0.37, 1.18	0.158	R	0.058	72.2	/	/
	African	1	1.00	0.52, 1.91	1.000	/	/	/	/	/
**VISFATIN** rs4730153 **allele**	Asian	2	0.58	0.41, 0.83	0.003	F	0.972	0	/	/
**recessive**	Asian	2	0.38	0.07, 2.26	0.289	F	0.667	0	/	/
**dominant**	Asian	2	0.57	0.39, 0.83	0.004	F	0.946	0	/	/
**VISFATIN** rs16872158 **allele**	Asian	2	1.84	1.26, 2.68	0.001	F	0.724	0	/	/
**recessive**	Asian	2	1.34	0.22, 8.14	0.752	F	0.833	0	/	/
**dominant**	Asian	2	1.94	1.31, 2.89	0.001	F	0.747	0	/	/

*Abbreviations*: *R* random effects model, *F* fixed effects model

### Association between ADIPOQ rs1501299 polymorphism and knee OA

The included studies focusing on rs1501299 showed no significant heterogeneity in both the overall analysis and subgroup analyses leveled by population groups in all the models. Therefore, all the models were analyzed by the fixed effects model. None of the models showed any significant association in the overall analysis (allele model: OR = 1.10, 95% CI 0.96–1.26, *P* = 0.184; recessive model: OR = 1.27, 95% CI 0.92- 1.75, *P* = 0.142; dominant model: OR = 1.08, 95% CI 0.91–1.29, *P* = 0.376) (Fig. 2). Meanwhile, none of the models showed any significant association in subgroup analyses leveled by population groups either.

### Association between ADIPOQ rs2241766 polymorphism and knee OA

The included studies focusing on rs2241766 showed no significant heterogeneity in both the overall analysis and subgroup analyses leveled by population groups in all the models. Therefore, all the models were analyzed by the fixed effects model. None of the models showed any significant association in the overall analysis (allele model: OR = 1.11, 95% CI 0.92–1.34, *P* = 0.279; recessive model: OR = 0.97, 95% CI 0.65 -1.44, *P* = 0.861; dominant model: OR = 1.21, 95% CI 0.95–1.55, *P* = 0.125) (Fig. 3). In subgroup analyses, the rs2241766 polymorphism showed no significant association with knee OA in Europeans and Latin Americans, while statistically significant associations were observed in Asians in the dominant model (OR = 1.35, 95% CI 1.03–1.78, *P* = 0.028) (Fig. 3).

### Association between LEPR rs1137101 polymorphism and knee OA

All models were analyzed by the random effects model in both the overall analysis and subgroup analyses leveled by population groups, due to the existence of significant heterogeneity of rs1137101.

In the overall analysis, significant associations were observed in the recessive model (OR = 0.40, 95% CI 0.21–0.79, *P* = 0.008) (Fig. 4). In subgroup analyses, the rs1137101 polymorphism showed no significant association with knee OA in Europeans and Africans, while statistically significant associations were observed in Asians in the recessive model (OR = 0.43, 95% CI 0.19–0.96, *P* = 0.040) (Fig. 4).

### Association between VISFATIN rs4730153 polymorphism and knee OA

There is a two-stage study that examined the relationship between rs4730153 polymorphism and knee OA risk in Asians. The included studies focusing on rs4730153 showed no significant heterogeneity in all the analyses. Therefore, all the models were analyzed by the fixed effects model.

Significant associations were observed in the allele model (OR = 0.58, 95% CI 0.41–0.83, *P* = 0.003) and dominant model (OR = 0.57, 95% CI 0.39–0.83, *P* = 0.004) (Fig. 5).

### Association between VASFATIN rs16872158 polymorphism and knee OA

There is one study containing 2 stages that illustrated the relationship between rs16872158 polymorphism and knee OA risk in Asians. The included studies focusing on rs16872158 showed no significant heterogeneity in all the analyses. Therefore, all the models were analyzed by the fixed effects model.

Significant associations were observed in the allele model (OR = 1.84, 95% CI 1.26–2.68, *P* = 0.001) and dominant model (OR = 1.94, 95% CI 1.31–2.89, *P* = 0.001) (Fig. 6).

Publication bias.

**Fig. 7:**
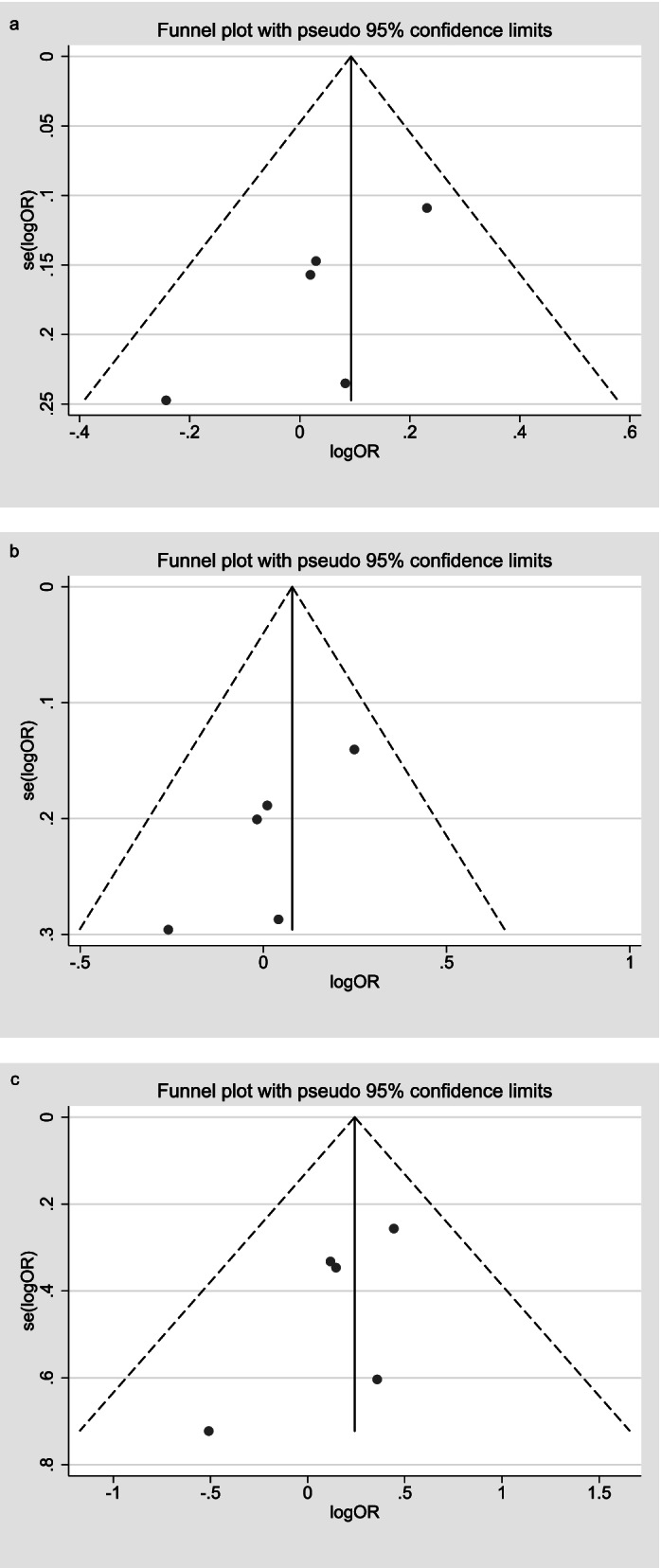
Funnel plot of publication bias for the association of ADIPOQ (rs1501299) polymorphism with OA in different genetic models. **a** allele model. **b** dominant model. **c** recessive model

No publication bias was evidenced in the included studies by the Egger’s test and Begg’s test. Table 3 and Fig. 7 presents the test results of publication bias on each gene polymorphism.

### Sensitivity analyses

In view of the significant heterogeneity in studies on the rs1137101 allele model, recessive model, and dominant model, sensitivity analyses were performed to examine the impact of any single study on the aggregate findings above based on the ORs with the matching 95% CIs before and after eliminating each study from the meta-analysis. The results remained consistent in all the models of gene polymorphisms above.

## Discussion

The present meta-analysis critically reviewed 5 studies for ADIPOQ rs1501299, 3 studies for ADIPOQ rs2241766, 3 studies for LEPR rs1137101, 2 studies for VISFATIN rs4730153 and 2 studies for VISFATIN rs16872158. Significant association was observed between LEPR rs1137101 and knee OA in the overall population and limited data revealed that associations may exist between ADIPOQ rs2241766 and knee OA in Asians, between VISFATIN rs4730153 and knee OA in Asians, and between VISFATIN rs16872158 and knee OA in Asians.

SNPs can affect OA phenotype through various mechanisms which may include.

post-translational modification of histones, non-coding RNAs, and DNA methylation [39], of which the latter is the most well-studied [40]. Studies of the cartilage DNA methylome have led to the discovery of OA-associated methylation quantitative trait loci (mQTLs), at which there is a correlation between genotype at an OA risk SNP and DNA methylation [13, 41–43]. Several OA risk loci have been identified which colocalise with genes encoding histone-modifying proteins [44–48]. The expression of these histone-modifying proteins is essential for cartilage homeostasis [39]. Meanwhile, OA risk SNPs have been identified in the region of cartilage-specific non coding RNAs that are known to be vital for homeostasis of the articular joint surface and are dysregulated in OA [41, 49].

Studies have been published on the associations of 2 SNPs (rs2241766 and rs1501299) in the ADIPOQ gene with knee OA, but the results were inconsistent. According to a study from Mexico, no association was observed between ADIPOQ rs2241766 and the risk of knee OA, but the effect of the interaction between polymorphisms ADIPOQ rs1501299 and PON1 rs662 seemed to play an important role in the knee OA pathogenesis. Two studies conducted by Honsawek et al. from Thailand found no association between ADIPOQ rs2241766 or rs1501299 polymorphism and the risk of knee OA [24, 25]. However, another two studies focusing on the Chinese population reported significant associations between ADIPOQ rs2241766 and rs1501299 polymorphisms and an increased risk of knee OA [21, 26]. These conflicting findings might be attributed to two reasons. The first one might be the genetic heterogeneity among different ethnicities, The second reason might be the varying sample sizes across different studies, which might introduce differences in data accuracy; moreover, a small sample size might even lead to false-

positive results. In the present meta-analysis, by investigating the associations of 2 SNPs (rs2241766 and rs1501299) with the risk of knee OA, we produced a hint that rs2241766 within the ADIPOQ gene might be a predisposing factor for the risk of knee OA in Asians.

Adiponectin, an adipocyte-derived hormone with multiple biological functions [50, 51], is traditionally considered as an anti-inflammatory adipokine in various disease states, including type 2 diabetes, nonalcoholic fatty liver disease and cardiovascular disease [52–57]. However, the role of adiponectin in the pathogenesis of OA remains controversial. On one hand, adiponectin was found to play a pro-

inflammatory role in OA. Adiponectin can stimulate the expression of interleukin-6 and matrix metalloproteinase-1/-3/-13, and the production of inducible nitric oxide synthase in both chondrocytes and OA synovium fluids through the mitogen-activated protein kinases, AdipoR1/5′-AMP-activated protein kinase, and the nuclear factor-

kappa B pathway, which may eventually lead to inflammation and matrix degradation in patients with OA [58–60]. On the other hand, serum adiponectin levels were found to be negatively associated with knee OA and synovial inflammation in destabilization of the medial meniscus and tibial fracture models [61, 62], indicating that adiponectin may play an anti-inflammatory role in OA. There were evidences supporting the existence of an association between rs2241766 and alterations of plasma adiponectin, especially in the Asian population [63–66]. Rs2241766, the exonic SNP, is a silent polymorphism which would not lead to changes in the sequence of amino acids [67]. However, evidence has been reported that many genes related to human diseases harbor exonic mutations could influence the pre-mRNA splicing [68]. In particular, the translationally silent mutations might inactivate genes by inducing the splicing machinery to skip mutant exons [68]. Consequently, SNP rs2241766 might affect the plasma level of adiponectin by affecting the splicing efficiency and/or accuracy of adiponectin mRNA [68]. Although the exact mechanism of rs2241766 modulation underlying OA susceptibility is still unclear, the data from the present study may provide a better understanding of its functional relevance to the pathogenesis of OA.

Two studies targeting at the Chinese population indicated an association between LEPR SNP and the susceptibility to knee OA; hence, it was speculated that there might be a genetic marker predicting the risk of this disease [27, 28]. On the contrary, Doudar et al. reported neither direct genetic association between rs1137101 SNP and the susceptibility of primary knee OA nor any gender difference in the frequency distribution of alleles or genotypes in Egyptians [30]. In the present meta-analysis, rs1137101 within the LEPR gene was found to be associated with a reduced risk of knee OA in the overall population and the Asian subgroup. Recently, due to the pro- inflammatory and pro-catabolic activities on the cartilage, the impaired leptin signal transduction was recognized as a new factor in the pathophysiology of OA [69]. Furthermore, due to the regulatory role of LEPR SNPs in the leptin signal pathway and the expression of LEPR in the cartilage [70, 71], LEPR was speculated to be a genetic risk factor for OA. By using arginine at codon 223 to replace the amino acid glutamine, the SNP rs1137101 in the LEPR gene represented a change in the extracellular domain of the LEPR protein, which would consequently result in structural changes in LEPR and potential alterations of the signaling capacity of leptin [72].

Visfatin is a multi-faceted, ubiquitous protein that acts on a number of diseases including OA [73, 74]. In accordance with a two-stage case–control study by Jiang et al. that examined the associations between 3 tagging polymorphisms in the VISFATIN gene and the risk of knee OA based on a sample containing 339 OA patients and 680 healthy subjects [32], the rs4730153 in VISFATIN appeared to be significantly associated with a reduced risk of knee OA, while the rs16872158 in VISFATIN was associated with an increased risk of knee OA in Chinese subjects. In view of that the genetic factors may be affected by different disease patterns, severities, genders and populations, more large-scale replication studies are needed to further verify the results related to SNPs on different population groups. It has been established that visfatin plays a role in the pro-inflammation process of OA. Meanwhile, 2 important mediators of cartilage destruction (i.e., IL-1β and lipopolysaccharide) in OA could enhance visfatin expression [75], which implied the existence of associations between inflammatory cytokines and visfatin [76]. Consistently, visfatin has been demonstrated to induce the expressions of IL-6 and monocyte chemoattractant protein 1 in chondrocytes and osteoblasts, implying a deleterious effect of this cytokine on OA [77]. Although evidence has been reported about the mechanism of visfatin underlying OA, there is a lack of evidence illustrating the mechanisms of rs4730153 and rs16872158 in affecting the expression of visfatin. In this regard, our study may provide a hint for detecting the pathogenesis of OA from a novel aspect.

Several limitations in this meta-analysis need to be highlighted. Firstly, some of the included studies did not match the confounding factors such as age, sex and BMI between case group and control group, and different factors for matching might increase the probability of residual confounding. Secondly, as the targeted populations were confined to Latin Americans and Asians, it is unclear whether the results could be generalized to other ethnic groups. Thirdly, there are only a small number of studies focusing on VISFATIN gene polymorphisms, and therefore, the statistical power was not strong enough to examine the associations between VISFATIN polymorphisms and OA. Fourthly, studies illustrated the associations between adipokines and OA mainly focus on knee OA, and there were limited data in hand or hip OA. Fifthly, genetic factors alone are unlikely to reliably identify individuals who will develop OA [5], as lifestyle changes (e.g., exercise, weight loss) may play a role in the development of OA [78]. Sixthly, candidate studies included in meta-analysis often lead to false-positive results due to lack of power, therefore, results should be interpreted with caution. Despite aforementioned limitations, our study is the first meta-analysis focusing on the associations between adipokines gene polymorphisms and the risk of knee OA, and may provide new insights into the etiology of knee OA.

## Conclusion

The present meta-analysis examined the potential associations between adipokines gene polymorphisms and knee OA. The association was observed in LEPR 1,137,101. Additionally, limited data revealed that associations may also exist in ADIPOQ rs2241766, VISFATIN rs4730153 and VISFATIN rs16872158. Further studies on different population groups with high quality and large sample size are needed to confirm and deepen our findings.

## Funding statement

This work was supported by the National Natural Science Foundation of China (81772413, 81702206), the National Key Research and Development Program of China (2018YFB1105705), the Xiangya Clinical Big Data System Construction Project of Central South University (45), the Central South University’s Innovation Key Foundation for Graduate (2018zzts256), and the Natural Science Foundation of Hunan Province (2017JJ3491, 2017JJ3492).

### Supplementary Information

**Additional file 1.**

## Data Availability

The data analysed during this study are included in the published articles and its supplementary information files.

## Appendix

### Search strategies

#### Pubmed:

1. osteoarthriti*[tiab] or osteoarthriti*[mh] or osteoarthro*[tiab] or gonarthriti*[tiab] or gonarthro*[tiab] or coxarthriti*[tiab] or coxarthro*[tiab] or osteo?arthritis[tiab]
2. polymorphism[tiab] or SNP*[tiab] or variant*[tiab] or mutation[tiab] or genotype[tiab] or allele[tiab] or haplogroup[tiab] or haplotype[tiab] or “genetic predisposition”[tiab] or “genetic susceptibility”[tiab] or “Polymorphism, Single Nucleotide”[MeSH]
3. ADIPOQ[tiab] or adiponectin[tiab] or “C1Q and collagen domain containing”[tiab] or ACDC[tiab] or ACRP30[tiab] or ADIPQTL1[tiab] or ADPN[tiab] or APM-1[tiab] or APM1[tiab] or GBP28[tiab]
4. LEP[tiab] or leptin[tiab] or OB[tiab] or OBS[tiab] or LEPD[tiab]
5. LEPR[tiab] or “leptin receptor”[tiab] or OBR[tiab] or OB-R[tiab] or CD295[tiab] or LEP-R[tiab] or LEPRD[tiab]
6. RETN[tiab] or resistin[tiab] or ADSF[tiab] or RSTN[tiab] or XCP1[tiab] or FIZZ3[tiab] or RETN1[tiab]
7. NAMPT[tiab] or “nicotinamide phosphoribosyltransferase”[tiab] or VF[tiab] or PBEF[tiab] or PBEF1[tiab] or VISFATIN[tiab] or 1110035O14Rik[tiab]
8. APLN[tiab] or apelin[tiab] or APEL[tiab] or XNPEP2[tiab]
9. CMKLR1[tiab] or “chemerin chemokine-like receptor 1”[tiab] or DEZ[tiab] or RVER1[tiab] or ChemR23[tiab] or CHEMERINR[tiab]
10. lipocalin-2[tiab] or LCN2[tiab] or p25[tiab] or 24p3[tiab] or MSFI[tiab] or NGAL[tiab]
11. or: 3–10
12. 1 and 2 and 11

### Embase:

1. (osteoarthriti* or osteoarthro* or gonarthriti* or gonarthro* or gonarthro* or coxarthriti* or coxarthro* or arthros* or arthrot*):ti,ab
2. (polymorphism or SNP* or variant* or mutation or genotype or allele or haplogroup or haplotype or “genetic predisposition” or “genetic susceptibility”):ti,ab or (“Polymorphism, Single Nucleotide”[MeSH])
3. (ADIPOQ or adiponectin or “C1Q and collagen domain containing” or ACDC or ACRP30 or ADIPQTL1 or ADPN or APM-1 or APM1 or GBP28):ti,ab
4. (LEP or leptin or OB or OBS or LEPD):ti,ab
5. (LEPR or “leptin receptor” or OBR or OB-R or CD295 or LEP-R or LEPRD):ti,ab
6. (RETN or resistin or ADSF or RSTN or XCP1 or FIZZ3 or RETN1):ti,ab
7. (NAMPT or “nicotinamide phosphoribosyltransferase” or VF or PBEF or PBEF1 or VISFATIN or 1110035O14Rik):ti,ab
8. (APLN or apelin or APEL or XNPEP2):ti,ab
9. (CMKLR1 or “chemerin chemokine-like receptor 1” or DEZ or RVER1 or ChemR23 or CHEMERINR):ti,ab
10. (lipocalin-2 or LCN2 or p25 or 24p3 or MSFI or NGAL):ti,ab
11. or: 3–10
12. 1 and 2 and 11

### Web of Science:

1. TOPIC:(osteoarthriti* or osteoarthro* or gonarthriti* or gonarthro* or gonarthro* or coxarthriti* or coxarthro* or arthros* or arthrot*)
2. TOPIC:(polymorphism or SNP* or variant* or mutation or genotype or allele or haplogroup or haplotype or “genetic predisposition” or “genetic susceptibility”) or (“Polymorphism, Single Nucleotide”[MeSH])
3. TOPIC:(ADIPOQ or adiponectin or “C1Q and collagen domain containing” or ACDC or ACRP30 or ADIPQTL1 or ADPN or APM-1 or APM1 or GBP28)
4. TOPIC:(LEP or leptin or OB or OBS or LEPD)
5. TOPIC:(LEPR or “leptin receptor” or OBR or OB-R or CD295 or LEP-R or LEPRD)
6. TOPIC:(RETN or resistin or ADSF or RSTN or XCP1 or FIZZ3 or RETN1)
7. TOPIC:(NAMPT or “nicotinamide phosphoribosyltransferase” or VF or PBEF or PBEF1 or VISFATIN or 1110035O14Rik)
8. TOPIC:(APLN or apelin or APEL or XNPEP2)
9. TOPIC:(CMKLR1 or “chemerin chemokine-like receptor 1” or DEZ or RVER1 or ChemR23 or CHEMERINR)
10. TOPIC:(lipocalin-2 or LCN2 or p25 or 24p3 or MSFI or NGAL)
11. or: 3-10
12. 1 and 2 and 11

## Abbreviations

OA: Osteoarthritis
SNPs: Single nucleotide polymorphisms
NOS: Newcastle–Ottawa Scale
ORs: Odd ratios
CIs: Confidence intervals
NA: Not available
PCR: Polymerase chain reaction
PCR–RFLP: Polymerase chain reaction and restriction fragment length polymorphism
R: Random effects model
F: Fixed effects model
